# Which biological and self-report measures of cannabis use predict cannabis dependency and acute psychotic-like effects?

**DOI:** 10.1017/S003329171800226X

**Published:** 2018-09-04

**Authors:** H. Valerie Curran, Chandni Hindocha, Celia J. A. Morgan, Natacha Shaban, Ravi K. Das, Tom P. Freeman

**Affiliations:** 1 Clinical Psychopharmacology Unit, University College London, Gower St, London, UK; 2 Department of Psychology, University of Exeter, Washington Singer Building, Perry Road, Exeter, UK; 3 National Addiction Centre, Institute of Psychiatry, Psychology & Neuroscience, King's College London, UK

**Keywords:** Biological markers, cannabinoids, cannabis, predictors of dependence, predictors of psychotic-like, self-report measures

## Abstract

**Background:**

Changes in cannabis regulation globally make it increasingly important to determine what predicts an individual's risk of experiencing adverse drug effects. Relevant studies have used diverse self-report measures of cannabis use, and few include multiple biological measures. Here we aimed to determine which biological and self-report measures of cannabis use predict cannabis dependency and acute psychotic-like symptoms.

**Method:**

In a naturalistic study, 410 young cannabis users were assessed once when intoxicated with their own cannabis and once when drug-free in counterbalanced order. Biological measures of cannabinoids [(Δ_9_*-tetrahydrocannabinol* (THC), *cannabidiol* (CBD), *cannabinol* (CBN) and their metabolites)] were derived from three samples: each participant's own cannabis (THC, CBD), a sample of their hair (THC, THC-OH, THC-COOH, CBN, CBD) and their urine (THC-COOH/creatinine). Comprehensive self-report measures were also obtained. Self-reported and clinician-rated assessments were taken for cannabis dependency [Severity of Dependence Scale (SDS), DSM-IV-TR] and acute psychotic-like symptoms [Psychotomimetic State Inventory (PSI) and Brief Psychiatric Rating Scale (BPRS)].

**Results:**

Cannabis dependency was positively associated with days per month of cannabis use on both measures, and with urinary THC-COOH/creatinine for the SDS. Acute psychotic-like symptoms were positively associated with age of first cannabis use and negatively with urinary THC-COOH/creatinine; no predictors emerged for BPRS.

**Conclusions:**

Levels of THC exposure are positively associated with both cannabis dependency and tolerance to the acute psychotic-like effects of cannabis. Combining urinary and self-report assessments (use frequency; age first used) enhances the measurement of cannabis use and its association with adverse outcomes.

## Introduction

Changes in the regulation of cannabis for recreational as well as medical use are currently continuing apace in many parts of the world. How patterns of use will subsequently change is not known, but even a small percentage increase in the current 183 million users worldwide will mean a considerable surge in absolute numbers. Quantifying the relative adverse and beneficial effects of cannabis and its constituent cannabinoids is, therefore, an important research priority (Curran *et al*., 2016). Cannabis use is associated with a 2-fold increased risk of developing a psychotic disorder (Marconi *et al*., 2016). Less attention has been paid to the much more common problem of cannabis addiction. It is estimated that 31% of past year cannabis users in the USA meet DSM-IV criteria for abuse or dependence (Hasin *et al*., 2015). However, the majority will be resilient and use cannabis without incurring serious mental health harms.

What predicts an individual's vulnerability or resilience to experiencing the harmful effects of cannabis? Several factors are currently thought to be important including early adolescent initiation of use (Coffey *et al*., 2003; Mokrysz *et al*., 2016), genetic factors (Di Forti *et al*., 2012; Morgan *et al*., 2016), concurrent tobacco use (Hindocha *et al*., 2015) and frequent (especially daily) cannabis use (Coffey *et al*., 2003; Chen *et al*., 2005). Other factors that may be important include the level of Δ_9_-tetrahydrocannabinol (THC) and other cannabinoids – especially cannabidiol (CBD) – in the strains that individuals use (Morgan and Curran, 2008; Morgan *et al*., 2010; Di Forti *et al*., 2015; Curran *et al*., 2016; Freeman *et al*., 2018).

One impediment to drawing conclusions about these risk factors is the varying measures of cannabis use that different studies employ. Despite growing international interest in this issue, there are currently no agreed standardised measures for assessing cannabis use in research (Yücel *et al*., 2016; Hindocha *et al*., 2017; Kögel *et al*., 2017). Although the majority of studies employ self-report measures (e.g. frequency of use; years used) few include questions on potency, dose and strain of cannabis (van der Pol *et al*., 2014). A minority employ biological measures, and when these are used there is much diversity in both the types of samples taken (e.g. hair, saliva, plasma, urine, actual cannabis used) and analyses subsequently carried out. Most estimate levels of Δ_9_−tetrahydrocannabinol (THC) and/or its metabolites, sometimes also cannabidiol (CBD) and less often other cannabinoids (Morgan and Curran, 2008; Demirakca *et al*., 2011; Freeman *et al*., 2014; Yücel *et al*., 2016).

If we could predict which variations in measures of cannabis use are and are not associated with adverse effects, then this would inform harm-reduction strategies which in turn would benefit those using cannabis for either recreational or medicinal purposes. Further, there are pragmatic reasons to explore which measures may be more or less associated with adverse outcomes because biological measures can be seen as personally intrusive and samples can be expensive to analyse.

We, therefore, set out to explore associations between multiple measures of cannabis use and two types of outcomes: the main harm we focussed on was dependence on cannabis; we also investigated acute psychotic-like effects that individuals experienced after ingesting the drug. Each of these outcomes was assessed by both self-report and by clinician-ratings. Three types of biological measures were used: analyses of cannabinoids (THC, CBD) and related metabolites in (i) participants’ hair, (ii) their urine and (iii) samples of cannabis each had used acutely. Self-report measures of use were: age of onset, years used, amount, frequency, time to smoke 3.5 g, the amount spent per week on buying cannabis, time since last use and preference or not for high potency cannabis strains. Our aim was to determine which measure or combination of measures best predicted the two outcomes.

We hypothesised firstly that using cannabis more frequently and using high (as opposed to low) potency varieties would be associated with increased rates of cannabis dependency (Morgan *et al*., 2010; Freeman and Winstock, 2015; Freeman *et al*., 2018). Secondly, we hypothesised that the use of high potency strains would lead to more acute psychotic-like experiences than the use of low potency strains (Di Forti *et al*., 2015). Thirdly, we hypothesised that CBD might mitigate the harmful effects of THC on both cannabis dependence and acute psychosis-like symptoms (Morgan and Curran, 2008; Bhattacharyya *et al*., 2010; Morgan *et al*., 2010; Englund *et al*., 2013).

## Method

### Design & participants

A repeated measures design was used whereby participants were tested on two separate days 7 ± 2 days apart in their own homes, once when acutely intoxicated with their own cannabis and once when non-intoxicated (Morgan *et al*., 2016). Session order was counterbalanced so that approximately 50% of participants were intoxicated on the first test day, non-intoxicated on the second, and 50% vice versa.

Inclusion criteria were: aged 16–24 years, fluent English, using cannabis at least once a month for at least a year, no learning impairments, no clinical diagnosis of a substance use disorder and no history of, or current, psychotic illness. We aimed to recruit 200 daily users of cannabis and 200 ‘recreational’ users (using less than daily). Regular users (>once/month) of any drug except cannabis, tobacco or alcohol were excluded.

Participants were recruited via advertisement and word of mouth. In all 410 completed the study, of whom 194 reported daily use and 216 less frequent use. This study was approved by UCL's Research Ethics Committee.

## Procedure

Participants gave written, informed consent on both testing days and were paid at the end of the second day. They were texted 24 h before each testing day to remind them to abstain from using alcohol or other drugs from then until after testing had finished. On the intoxicated test day (before testing), participants gave a urine sample. Prior to testing they also provided a 0.3 g sample of the cannabis they were about to smoke and testing began immediately after the participant had finished smoking it. On the non-intoxicated day, a hair sample was taken from each participant of which 3 cm (from the scalp) was analysed. Researchers were fully trained to administer and score clinician-rated as well as other measures.

## Assessments

### Outcome variables

*Cannabis dependence:* The *Severity of Dependence Scale* (*SDS*) was used to provide a self-report measure of psychological aspects of cannabis dependence (Gossop *et al*., 1995; Piontek *et al*., 2008). SDS is scored out of 15 and a cut-off of 3+ has been used for indexing cannabis dependence (Swift *et al*., 1998; Martin *et al*., 2006). Clinical diagnosis of cannabis dependence was based on *DSM-IV-TR criteria* (American Psychiatric Association, 2000): a maladaptive pattern of cannabis use leading to clinically significant impairment or distress, manifested by at least three of the six criteria within the last 12 months.

*Psychotic-like symptoms*: self-rated symptoms were assessed with the *Psychotomimetic States Inventory* (*PSI*; Mason et al., 2008) which was completed on both test days. An abridged version of the *Brief Psychiatric Rating Scale* (*BPRS*) (Krystal et al., 1994; Ventura et al., 2000) was used as the clinician-rated measure of psychotic-like symptoms. On both scales, a change score was calculated (score on intoxicated minus score on the non-intoxicated day) and used as the measures of acute psychotic-like symptoms.

### Predictor variables

#### Biological variables

*Cannabinoids in cannabis samples:* The 0.3 g sample of the cannabis that each participant provided was analysed for concentrations (% of sample weight) of THC (THC and THC acid) and of CBD (CBD and CBD acid) by gas chromatography coupled to mass spectrometry (Forensic Science Service, Birmingham).

*Urine:* urine collected on the intoxicated day was analysed for THC-COOH concentrations (limit of quantification: 2 ng/ml). These were corrected for creatinine concentrations (mg/ml) and are expressed as THC-COOH/creatinine (ng/mg); Alere, UK.

*Hair:* the hair sample was a 3 cm segment cut from the scalp of each participant on the non-intoxicated day. Gas chromatography coupled to mass spectrometry was used to determine CBD, THC, CBN, THC:COOH, and THC:OH (Alere, UK). Consistent with previous studies (Morgan and Curran, 2008; Yücel *et al*., 2016) and with research indicating lack of valid quantification of cannabis use by hair (Taylor *et al*., 2017) samples were classified according to whether each analyte was present or absent.

#### Self-report variables

*Drug history:* a comprehensive cannabis use history was taken including age cannabis use first started, years used, days per month used, the time taken to smoke 1/8th ounce (3.5 g), time since last use and money spent on cannabis per week. Participants additionally completed a modified Cannabis Experience Questionnaire (Barkus *et al*., 2006). This included questions about participants’ preference (yes/no) for high potency cannabis (skunk, sensimilla) as opposed to other strains (e.g. resin/hash, low potency herbal).

*Demographic variables:* Age and Sex data were recorded at screening.

### Statistical analyses

Analyses were conducted in Stata/IC *v.* 15.1 (StataCorp, College Station, TX) and SPSS v.23 (IBM Corp, Armonk, NY). DSM-IV-TR diagnosis of cannabis dependence was coded as 0 or 1 (dependent, not dependent; not dependent was the reference group). SDS was non-normally distributed with a negative skew that could not be corrected by transformation, therefore it was coded as a dichotomous variable (dependent, not dependent coded 0 or 1) based on an adult cut-off of 3 (not dependent was the reference group). Hair cannabinoid/cannabinoid metabolites were coded as present or absent (0 or 1; absent was the reference group). Sex was coded as female or male (1 or 2; male was the reference group). Finally, preference for high potency cannabis was coded as present or absent (preference for other strains or no preference; coded as 0 or 1 with absent as the reference category). We correlated demographic covariates, biological and self-reported predictors with the four outcome variables. We used Pearson's product moment correlations (*r*) or Spearman's rho (*r*_s_) for correlations between continuous variables, point biserial correlation (*r*_pb_) for correlations between dichotomous and continuous variables and the Phi coefficient (*r*_*φ*_) for the association between two dichotomous variables (this was chosen over chi-square as it accounts for the number of participants and is a measure of effect size). Predictors were included in the model-fitting process if they were significant correlates at a conservative *α* threshold of ⩽0.001. For each outcome variable, we entered all correlated variables into forward-fit linear models (binary logistic models for SDS and DSM-IV-TR) starting with the null model. We chose mixed effects modelling approach using maximum likelihood estimation in order to fit both fixed and random effects as appropriate, and for improved handling of missing data compared with generalised linear model approaches. Candidate variables selected from the correlational analysis (*p* ⩽ 0.001) were added as fixed effects. Variables were retained in the model if they significantly improved model fit as assessed by a lower Bayesian Information Criteria (BIC) and a significant (*α* ⩽ 0.05) Likelihood Ratio Test. We chose to use the BIC over other goodness-of-fit statistics as it penalises model complexity, protecting against overfitting. Variable entry order was determined with univariate BIC values with lower BIC values representing better model fit (online Supplementary Table 2). The final mixed effects models were checked for violation of assumptions. Multicollinearity was not a concern in any model (all VIFs ⩽ 1.76). A correlation matrix between self-report and biological predictor variables can be found in Supplementary Table 3. In all final models, a random intercept for ‘participant’ was added to investigate if it improved model fit. The unstructured variance-covariance structure was selected. Random effects parameters did not improve model fit and were non-significant in all models, did not change the pattern of results, and therefore were not included in any of the final models.

## Results

### Sample characteristics

In all, 410 individuals completed the study, they were aged M (s.d.) 20.56 (1.68) years and 27% (113) were female. Participants’ mean age of first cannabis use was 14.94 (2.05) years, they had been using cannabis on average for 4.88 (2.36) years and used it 16.87 (10.95) days per month. They had last used cannabis 4.20 (7.24) days previously. Participants took 10.31 (17.25) days to smoke 3.5 g (one-eighth of an ounce) of cannabis and spent £20.26 (£22.64) per week on the drug. Full descriptive statistics are in Supplementary Table 1 which specifies the number of participants for whom data on each variable was available. Some hair samples were not heavy enough for analysis leaving 344 which were fully analysed; for cannabis donated prior to ingestion full analyses were successfully conducted on 366 samples (see online Supplementary Table 1 for full details).

#### Outcome variables

*Cannabis dependence*: 43.7% of participants met the cut-off for cannabis dependence on the SDS and 48.4% met DSM-IV-TR criteria for cannabis dependence.

**Fig. 1.**
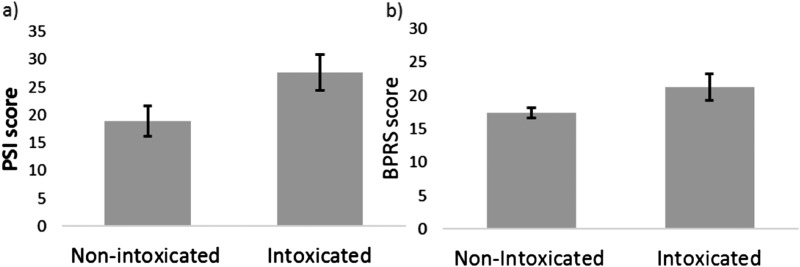
Cannabis increased psychotic-like symptoms as measured by (a) self-report on the PSI, Psychotomimetic States Inventory; (b) clinical assessment on the BPRS, Brief Psychiatric Rating Scale. Error bars are 95% confidence intervals.

*Psychotic-like symptoms:* Cannabis robustly increased psychotic-like symptoms according to both self-report assessment on the PSI (*t*_383_ = 10.067, *p* < 0.001, *d* = 0.516) and clinical assessment on the BPRS (*t*_401_ = 7.857, *p* < 0.001, *d* = 0.413). The mean increase in psychotic-like symptoms was 8.61 (16.83) for the PSI and 3.91 (9.97) for the BPRS (Fig. 1).

### Biological and self-report measures of cannabis use: associations with outcome variables

#### Cannabis dependence: self-reported (SDS)

Cannabis dependence (SDS) was positively correlated with THC-COOH/creatinine in urine (*r*_*pb*_(345) = 0.350, *p* ⩽ 0.001), THC in hair (*r*_*φ*_(412) = 0.206, *p* ⩽ 0.001), CBD in hair (*r*_*φ*_(412) = 0.178, *p* ⩽ 0.001), CBN in hair (*r*_*φ*_(412) = 0.215, *p* ⩽ 0.001) THC-COOH in hair (*r*_*φ*_(412) = 0.170, *p* ⩽ 0.001), THC-OH in hair (*r*_*φ*_(412) = 0.135, *p* = 0.006), days per month of cannabis use (*r*_*pb*_(398) = 0.486, *p* ⩽ 0.001), preference for high potency cannabis (*r*_*φ*_(412) = 0.212, *p* ⩽ 0.001) and the amount of money spent on cannabis per week (*r*_*pb*_(411) = 0.412, *p* ⩽ 0.001). Cannabis dependence (SDS) was negatively correlated with last use of cannabis (*r*_*pb*_(401) = −0.244, *p* ⩽ 0.001) and time to smoke 3.5 g (*r*_*pb*_(392) = −0.199, *p* ⩽ 0.001). Univariate BIC values ranked in order (from lowest to highest) are shown in Supplementary Table 2. In the final model cannabis dependence indexed by the SDS was positively associated with both THC-COOH/creatinine in urine and days per month of cannabis use (Table 1).

**Table 1.** Associations between biological and self-report measures of cannabinoid exposure and cannabis dependence on the SDS

	OR	s.e.	95% CI		*z*	*p*
			Lower	Upper		
**THC-COOH/creatinine**	**1.004**	**0.001**	**1.0010**	**1.0060**	**3.0110**	**0.0026**
**Days per month of cannabis use**	**1.104**	**0.017**	**1.0710**	**1.1390**	**6.3050**	**0.0000**
Days to smoke 3.5 g of cannabis	1.000	0.011	0.9800	1.0210	0.0010	0.9991
Constant	0.105	0.037	0.0530	0.2090	−6.4430	0.0000

Bold significant associations at *p* < 0.05.

#### Cannabis dependence: clinician-rated (DSM-IV-TR)

Cannabis dependence (DSM–IV-TR) was associated with greater THC-COOH/creatinine in urine (*r*_*pb*_(344) = 0.228, *p* < 0.001), more days per month of cannabis use (*r*_*pb*_(397) = 0.431, *p* ⩽ 0.001), time to smoke 3.5 g (*r*_*pb*_(391) = −0.208, *p* ⩽ 0.001), a preference for high potency cannabis strains (*r*_*φ*_(411) = 0.165, *p* = 0.001) and more amount of money spent on cannabis per week (*r*_*pb*_(410) = 0.345, *p* ⩽ 0.001). Univariate BIC values ranked in order (from lowest to highest) are shown in Supplementary Table 2. In the final model, DSM-IV-TR diagnosis of cannabis dependence was positively associated with days per month of cannabis use (Table 2).

**Table 2.** Associations between biological and self-report measures of cannabinoid exposure and cannabis dependence (DSM-IV-TR)

DSM-IV-TR (*n* = 331)	OR	s.e.	95% CI		*z*	*p*
			Lower	Upper		
THC-COOH/creatinine	1.000	0.001	0.999	1.002	0.441	0.659
**Days per month of cannabis use**	**1.068**	**0.016**	**1.036**	**1.101**	**4.260**	**0.000**
Money spent on cannabis per week	1.016	0.010	0.997	1.035	1.650	0.099
Constant	0.207	0.050	0.130	0.332	−6.555	0.000

Bold significant associations at *p* < 0.05.

#### Acute psychotic-like symptoms: self-reported (PSI)

Self-reported psychotic-like symptoms (PSI) correlated positively with both THC-COOH/creatinine in urine (*r*(324) = −0.196, *p* *<* 0.001) and age of first cannabis use (*r*(384) = 0.206, *p* *<* 0.001). Univariate BIC values ranked in order (from lowest to highest) are shown in Supplementary Table 2. In the final model (Table 3) cannabis-induced psychotic-like symptoms were positively associated with age of first cannabis use, and negatively associated with THC-COOH/creatinine in urine.

**Table 3.** Associations between biological and self-report measures of cannabinoid exposure and cannabis-induced psychotic-like symptoms on the PSI

	B	s.e.	95% CI		z	*p*
			Lower	Upper		
**Age of first cannabis use**	**1.588**	**0.433**	**0.738**	**2.437**	**3.664**	**0.0002**
**THC-COOH/creatinine**	**−0.011**	**0.003**	**−0.017**	**−0.004**	**−3.131**	**0.0017**
Constant	−13.801	6.587	−26.711	−0.891	−2.095	0.0361

Bold significant associations at *p* < 0.05.

#### Acute psychotic-like symptoms: clinician-rated (BPRS)

Clinician-rated psychotic-like symptoms (BPRS) correlated negatively with age (*r*(400) = −0.127, *p* = 0.011), THC in hair (*r*_*pb*_(402) = −0.135, *p* = 0.007), CBD in hair (*r*_*pb*_(402) = −0.123, *p* = 0.013) and positively with CBN in hair (*r*_*pb*_(402) = 0.116, *p* = 0.020). None of these variables met the criteria for inclusion in the final model (*α* ⩽ 0.001).

## Discussion

The findings of this study provide converging evidence that a combination of biological and self-report measures provides the best model fit for predicting both dependence and acute psychotic-like effects of cannabis in healthy young cannabis users.

Cannabis dependence was most strongly predicted by the number of days per month that the individual used the drug, with the greater frequency associated with greater dependency. This was the case for both self-ratings (SDS) and clinician-ratings (DSM-IV-TR) and was hypothesised based on the findings of many previous studies (Curran *et al*., 2016). In addition, on the self-rating measure, level of THC-COOH/creatinine in urine improved model fit, with higher urinary levels predicting increased dependency. In terms of specific cannabinoids, the importance of the level of the THC metabolite in predicting dependence supports the hypothesis that higher levels of THC ingested are associated with increased risk of dependency (Curran *et al*., 2016; Freeman *et al*., 2018). The average THC content of cannabis samples was approximately 10% and ranged widely. In contrast, average CBD content was less than 1% with a much narrower range of values. This reflects the current cannabis market in many countries with a predominance of high potency ‘skunk’. Indeed, Potter *et al*. (2018)’s recent analysis of UK police seizures found that 94% of strains were completely lacking in CBD. CBD was detected in the hair of a third of our participants but was not predictive/protective of dependence or psychotic-like symptoms. These findings suggest a clear role for higher THC levels predicting dependency whilst the near absence of CBD suggests we cannot evaluate the role it plays from our data.

In terms of acute psychotic-like effects, again a combination of biological (urine) and self-report (age of first use) measures provided the optimal model fit on the self-rated measure (PSI). Quantitative measurements of THC-COOH/creatinine in urine samples taken before using cannabis on the intoxicated day were again a strong predictor. However, the association was negative, such that lower levels in urine when sober predicted a greater increase in psychotic-like symptoms when acutely intoxicated with cannabis. This strongly suggests a tolerance effect whereby lower levels of THC-COOH/creatinine would reflect less (at least) recent use of cannabis and a resulting stronger effect of the drug. Indeed, there were very strong correlations between THC-COOH/creatinine and days since last use of cannabis (*r* = −0.57; 32% shared variance) and days used per month (*r* = 0.68; 46% shared variance). This supports our previous findings that a greater increased in psychotic-like symptoms (again measured with the PSI) was associated with the less frequent use of cannabis (Mason *et al*., 2008; Morgan *et al*., 2012). D'Souza *et al*. (2008) found similar tolerance effects to the psychotomimetic effects of THC in frequent cannabis users and similar tolerance has been reported for the acute cognitive-impairing effects of cannabis (Ramaekers *et al*., 2009).

It is likely that the same tolerance mechanism explains why the age of first use was positively associated with a greater increase in psychotic-like symptoms (PSI) from the sober to the acutely intoxicated state. Being older at first use of cannabis predicted higher levels of acute psychotic-like symptoms, suggesting that those individuals who started using at a younger age had developed greater tolerance to these acute effects of the drug.

No significant predictors emerged for clinician ratings of psychotic-like effects (BPRS). This measure was sensitive to the acute effects of cannabis showing a very significant increase in scores when participants were intoxicated. The increase in psychotic-like effects on the BPRS also correlated positively with those in the PSI. At the same time the variance of scores on the BPRS was much less than on the PSI which may have mitigated against the sensitivity of the BPRS as an outcome. Further, the self-rating measure was specifically developed to be sensitive to the psychotomimetic effects of drugs like cannabis and ketamine so its greater sensitivity than a brief clinician screening tool is unsurprising.

### Which self-report and biological variables are most important for inclusion in future studies?

The two key variables of urinary THC-COOH/creatinine and self-reported frequency of use were stronger predictors than other measures of cannabis use. On the basis of our findings, these two measures are most important for measuring cannabis use and its consequences, and should be prioritised in future research studies such as observational studies investigating cannabis harms, as well as randomised controlled trials aiming to reduce levels of cannabis use. It is important to note that in addition to the urinary data, objective measures of cannabinoids and their metabolites in other biological samples (users’ own cannabis, samples of hair) were modestly associated with self-report measures of cannabis use, as well as measures of harm in exploratory correlations. Although these measures did not emerge as strong predictors of either dependence or acute psychotic-like effects, they may be useful adjunctive measures or to test specific hypotheses [e.g. CBD in hair as a measure of long-term protection from harm (Morgan and Curran, 2008; Demirakca *et al*., 2011; Yücel *et al*., 2016)]. Here we did not find a protective effect of CBD measured in hair which may well reflect the near negligible levels of CBD in UK cannabis (Potter *et al*., 2018). Further, we did not have a measure for CBD metabolites in urine which could have provided a more accurate measure of dose of CBD consumed.

Strengths of this study include a sample of over 400 young cannabis users and high levels of dependency (over 43%) in the sample, which reflected our deliberate recruiting of daily as well as recreational users. Further, we obtained very significant acute psychotic-like effects of cannabis on the intoxicated day. This we used as an index of clinically relevant changes, as a study of psychotic disorder would require much larger sample sizes including clinically diagnosed populations. Our findings that early onset of cannabis use predicted less transitory acute psychotic-like effects contrasts with epidemiological findings of early-onset increasing the risk of developing a psychotic disorder (Di Forti *et al*., 2014) although they are consistent with evidence from experimental studies (D'Souza *et al*., 2008). A limitation endemic to naturalistic studies was that we had limited information of the dose of THC that participants ingested. A further limitation was that biological analyses were available for 89% of cannabis, 85% of urine and 84% of hair samples rather than the full 100% (410 participants). Finally, due to the cross-sectional design of this study, we are unable to establish the existence or direction of causality. Other potential measures of cannabis which may be useful to employ include objectively quantifying cannabis in preparations such as ‘joints’ rolled (Hindocha *et al*., 2017; Kögel *et al*., 2017; Hindocha *et al*., 2018).

In summary, key measures of cannabis use which are positively predictive of increased drug dependence are frequency (days per month used) and a quantitative measure of urinary THC:COOH/creatinine. The same urinary THC:COOH/creatinine measure also negatively predicted acute psychotic-like effects, alongside self-reported age of first cannabis use. Most biological variables, although positively correlated with outcome measures, were not strong predictors of outcomes. In this context, costly analyses of hair and cannabis samples appear less important in predicting dependence or transitory psychotic-like effects. These findings could usefully inform future studies needing relevant measures of cannabis use, for example, the longitudinal ABCD study (Lisdahl *et al*., 2018), which currently includes measurement of cannabinoids in hair but does not include a quantitative index of THC:COOH/creatinine in urine.

As cannabis now stands poised to join alcohol and tobacco as a legal drug in many parts of the world, the prediction of who is resilient or vulnerable to its harms is increasing in importance. In terms of cannabis dependence, our findings suggest the optimal harm reduction strategy is to reduce the frequency of using the drug. In terms of measures, this study strongly implies that future studies generally include a self-report measure of days per month used and urinary THC-COOH/creatinine levels.

## Supplementary material

For supplementary material accompanying this paper visit https://doi.org/10.1017/S003329171800226X. click here to view supplementary material
