# Supplementary material for: Which biological and self-report measures of cannabis use predict cannabis dependency and acute psychotic-like effects?
Source: Psychol Med. 2018 Sep 4;49(9):1574–80. doi: 10.1017/S003329171800226X (PMC6541869; doi:10.1017/S003329171800226X)
Supplement: Supplementary file 1 [file S003329171800226Xsup001.docx]

SUPPLEMENTARY MATERIALS

What biological and self-report measures of cannabis use predict cannabis dependency and acute psychotic-like effects?

H Valerie Curran^1^, Chandni Hindocha^1^,Celia Morgan^1,2^, Natacha Shaban^1^,

Ravi Das^1^ & Tom P Freeman^1^

1. Clinical Psychopharmacology Unit, University College London, Gower St, London, UK.

2. Department of Psychology, University of Exeter, Washington Singer Building, Perry Road, Exeter, UK.

**Correspondence to:** Prof H Valerie Curran: [v.curran@ucl.ac.uk](mailto:v.curran@ucl.ac.uk)

Supplementary Table 1: Self-report and biological indices of cannabinoid exposure, and their associations with cannabis dependence (Severity of Dependence Scale; SDS and DSM-IV-TR) and cannabis-induced psychotic symptoms (Psychotomimetic States Inventory; PSI and Brief Psychiatric Rating Scale; BPRS)

|  |  | |  | **Cannabis Dependence** | | **Cannabis-induced psychotic symptoms** | |
| --- | --- | --- | --- | --- | --- | --- | --- |
|  | **N** | | **Mean (SD)** | **SDS** | **DSM IV-TR** | **PSI** | **BPRS** |
| SDS (No/Yes) | 412 | | 43.7% yes | - | **0.381*****^b^ | -0.036^a^ | -0.023 ^a^ |
| DSM IV criteria (No/Yes) | 411 | | 48.4% yes |  | - | -0.060^a^ | -0.068 ^a^ |
| Change PSI | 384 | | 8.65 (16.83) |  |  | - | **0.131****^c^ |
| Change BPRS | 402 | | 3.91 (9.97) |  |  |  | - |
| Age | 416 | | 20.56 (1.67) | 0.077^a^ | -0.004^a^ | -0.032^c^ | **-0.127***^c^ |
| Sex (M/F) | 418 | | 113 Female | -0.023^b^ | -0.020^b^ | 0.019 ^a^ | 0.224^a^ |
| THC % in cannabis | 366 | | 9.60 (4.69) | -0.010 ^a^ | -0.049^a^ | -0.083^c^ | -0.028^c^ |
| CBD % in cannabis | 366 | | 0.82 (1.66) | 0.009 ^a^ | 0.053^a^ | 0.037^c^ | -0.067^c^ |
| Urine THC-COOH/creatinine | 348 | | 122.41 (253.21) | **0.350*****^a^ | r=0.228*** | **-0.196*****^c^ | -0.078^c^ |
| Hair-THC (no/yes) | 412 | | 55.7% yes | **0.206*****^b^ | 0.094^b^ | -0.094 ^a^ | **-0.135**** ^a^ |
| Hair-CBD (no/yes) | 412 | | 33.5% yes | **0.178*****^b^ | 0.074^b^ | -0.071 ^a^ | **-0.123*** ^a^ |
| Hair-CBN (no/yes) | 344 | | 48.1% yes | **0.215*****^b^ | 0.030^b^ | **-0.102*** | **-0.116*** ^a^ |
| Hair-THC-COOH (no/yes) | 344 | | 25.6% yes | **0.170*****^b^ | **0.136****^b^ | -0.068 ^a^ | -0.087 ^a^ |
| Hair-THC-OH (no/yes) | 344 | | 24.9% yes | **0.135****^b^ | 0.069^b^ | -0.021 ^a^ | -0.072 ^a^ |
| Age of first use (years) | 413 | | 14.94 (2.05) | -0.033^a^ | **-0.122***^a^ | **0.206***^c^** | 0.032^c^ |
| Last used (days) | 401 | | 4.20 (7.24) | **-0.244*****^a^ | -0.079^a^ | 0.162****^d^** | 0.069^d^ |
| Years Used (years) | 401 | | 4.88 (2.36) | 0.038 ^a^ | -0.093^a^ | **-0.148****^c^ | -0.039^c^ |
| Days per month (days) | 398 | | 16.87 (10.95) | **0.486*****^a^ | **0.431***** ^a^ | **-0.141****^d^ | -0.049^d^ |
| Time to smoke 3.5g (days) | 392 | | 10.31 (17.25) | **-0.199*****^a^ | **-0.208*****^a^ | **0.143****^d^ | -0.023^d^ |
| Potency Preference (No/Yes) | 413 | | 39% yes | **0.212*****^b^ | **0.165*****^b^ | -0.076 ^a^ | -0.073 ^a^ |
| Money spent per week on cannabis(£) | 411 | | 20.26 (22.64) | **0.412*****^a^ | **0.345*****^a^ | **-0.144****^d^ | -0.020^d^ |
| ** p≤ .05, ** p≤ .01, *** p≤ .001*  ^a^ point biserial correlation, ^b^Phi correlation coefficient, ^C^ Peasons correlation coefficient ^d^ Spearmans rho | |  |  |  |  |  |  |

Supplementary Table 2: Univariate Bayesian Information Criterion (BIC) values ranked in order from lowest to highest.

| SDS | BIC | DSM-IV-TR | BIC | PSI | BIC |
| --- | --- | --- | --- | --- | --- |
| THC-COOH/creatinine | 414.686 | THC-COOH/creatinine | 465.757 | THC-COOH/creatinine | 2735.501 |
| Days per month (days) | 456.812 | Days per month (days) | 484.628 | Age of first cannabis use (years) | 3258.232 |
| Money spent per week on cannabis (£) | 490.646 | Money spent per week on cannabis (£) | 521.664 |  |  |
| Last used (days) | 511.722 | Time to smoke 3.5g (days) | 533.635 |  |  |
| Time to smoke 3.5g (days) | 529.820 | Potency Preference (No/Yes) | 570.152 |  |  |
| Hair-CBN (No/Yes) | 557.468 |  |  |  |  |
| Potency Preference (No/Yes) | 558.107 |  |  |  |  |
| Hair-THC (No/Yes) | 558.858 |  |  |  |  |
| Hair-CBD (No/Yes) | 563.581 |  |  |  |  |
| Hair-THC-COOH (No/Yes) | 564.838 |  |  |  |  |
| Hair-THC-OH (No/Yes) | 569.144 |  |  |  |  |

Supplementary table 3: Associations between self-report and biological indices of cannabinoid exposure.

|  | Age | Sex | THC % in cannabis | CBD % in cannabis | THC:  creatinine ratio | Hair-THC | Hair-CBD | Hair-CBN | Hair-THC-COOH | Hair-THC-OH | Age of first use | Last used | Years Used | Days per month | Time to smoke 3.5g | Potency Preference | Money spent |
| --- | --- | --- | --- | --- | --- | --- | --- | --- | --- | --- | --- | --- | --- | --- | --- | --- | --- |
| Age | 1.000 |  |  |  |  |  |  |  |  |  |  |  |  |  |  |  |  |
| Sex (M/F) | -.033  ^a^ | 1.000 |  |  |  |  |  |  |  |  |  |  |  |  |  |  |  |
| THC % in cannabis | .065  ^c^ | -.066^a^ | 1.000 |  |  |  |  |  |  |  |  |  |  |  |  |  |  |
| CBD % in cannabis | .116*  ^c^ | -.037^a^ | -.036  ^c^ | 1.000 |  |  |  |  |  |  |  |  |  |  |  |  |  |
| THC:creatinine ratio | .043  ^c^ | -.005^a^ | .139*  ^c^ | -.058  ^c^ | 1.000 |  |  |  |  |  |  |  |  |  |  |  |  |
| Hair-THC (no/yes) | .071  ^a^ | -.043^b^ | .271***^a^ | -.014  ^a^ | .238***  ^a^ | 1.000 |  |  |  |  |  |  |  |  |  |  |  |
| Hair-CBD (no/yes) | .069  ^a^ | -.055^b^ | .165**  ^a^ | .076  ^a^ | .189***  ^a^ | .622***  ^b^ | 1.000 |  |  |  |  |  |  |  |  |  |  |
| Hair-CBN (no/yes) | .093  ^a^ | -.036^b^ | .236***^a^ | .051  ^a^ | .196***  ^a^ | .800***  ^b^ | .727***^b^ | 1.000 |  |  |  |  |  |  |  |  |  |
| Hair-THC-COOH (no/yes) | .013  ^a^ | -.048^b^ | .103  ^a^ | -.040  ^a^ | .262***  ^a^ | .523***  ^b^ | .617***^b^ | .489***^b^ | 1.000 |  |  |  |  |  |  |  |  |
| Hair-THC-OH (no/yes) | .032  ^a^ | -.014^b^ | .061  ^a^ | .013  ^a^ | .251***  ^a^ | .513***  ^b^ | .659***^b^ | .565***^b^ | .728***^b^ | 1.000 |  |  |  |  |  |  |  |
| Age of first use (years) | .183***  ^c^ | .039  ^a^ | .081  ^c^ | -.025  ^c^ | -.110*  ^c^ | .007^a^ | .009  ^a^ | -.013  ^a^ | -.003  ^a^ | .011  ^a^ | 1.000 |  |  |  |  |  |  |
| Last used (days) | -.007  ^d^ | .014  ^a^ | .060  ^d^ | .081  ^d^ | -.570***^d^ | -.062^a^ | -.053  ^a^ | -.810  ^a^ | .025  ^a^ | .013  ^a^ | .143**  ^d^ | 1.000 |  |  |  |  |  |
| Years Used (years) | .453***  ^c^ | -.072  ^a^ | -.087  ^a^ | .116*  ^c^ | .104  ^c^ | .042  ^a^ | .074  ^a^ | .070  ^a^ | -.004  ^a^ | .020  ^a^ | -.568***^c^ | -.065d | 1.000 |  |  |  |  |
| Days per month (days) | -.043  ^d^ | -.006  ^a^ | -.065  ^d^ | -.036  ^d^ | .683***  ^d^ | .234***  ^a^ | .194***^a^ | .227***^a^ | .225***^a^ | .188***^a^ | -.231***^d^ | -.666***^d^ | .099*  ^d^ | 1.000 |  |  |  |
| Time to smoke 3.5g (days) | .264***^d^ | .091  ^a^ | -.047  ^d^ | -.058  ^d^ | -.420***^d^ | -.212***^a^ | -.124*  ^a^ | -.153**  ^a^ | -.107*  ^a^ | -.063^a^ | .290***  ^d^ | .319***  ^d^ | 0.014  ^d^ | -.454***^d^ | 1.000 |  |  |
| Potency Preference (No/Yes) | -.008  ^a^ | -.021^b^ | .130*  ^a^ | -.029  ^a^ | .317***  ^a^ | .137**  ^b^ | .101*  ^b^ | .171***^b^ | .113*  ^b^ | .204***^b^ | -.148**  ^a^ | -.106*  ^a^ | .091  ^a^ | .300***  ^a^ | -.196***^a^ | 1.000 |  |
| Money spent per week on cannabis (£) | -.061  ^d^ | -.046^a^ | .042  ^d^ | -.036  ^d^ | .656***  ^d^ | .242***  ^a^ | .176***^a^ | .229***^a^ | .274***^a^ | .192***^a^ | -.205***^a^ | -.567***^d^ | .072  ^d^ | .788***  ^d^ | -.564***^d^ | .359***  ^a^ | 1.000 |

** p≤ .05, ** p≤ .01, *** p≤ .001*

^a^ point biserial correlation, ^b^Phi correlation ^C^ Peasons correlation ^d^ Spearmans rho
